# Acute Effects on Impact Accelerations Running with Objects in the Hand

**DOI:** 10.3390/life11060550

**Published:** 2021-06-11

**Authors:** Roberto Sanchis-Sanchis, Alberto Encarnación-Martínez, Jose I. Priego-Quesada, Inmaculada Aparicio, Irene Jimenez-Perez, Pedro Pérez-Soriano

**Affiliations:** 1 Research Group in Sports Biomechanics (GIBD), Department of Physical Education and Sports, University of Valencia, 46010 Valencia, Spain; roberto.sanchis@uv.es (R.S.-S.); alberto.encarnacion@uv.es (A.E.-M.); j.ignacio.priego@uv.es (J.I.P.-Q.); inmaculada.aparicio@uv.es (I.A.); i.jimenez.gibd@gmail.com (I.J.-P.); 2 Physical Education and Sport, University of Alicante, 03690 San Vicente del Raspeig, Spain

**Keywords:** impact accelerations, run, asymmetric handloads, spatio-temporal parameters

## Abstract

Amateur runners usually run carrying implements in their hands (keys, a mobile phone, or a bottle of water). However, there is a lack of literature about the effects of different handloads on impact accelerations. Thus, this study aimed to analyse the effects of carrying different objects in the hand on impact accelerations during running. Nineteen male recreational runners (age 24.3 ± 6.8 years, training volume of 25 ± 7.38 km/week) performed twenty minutes of running on a treadmill at 2.78 m/s with four different conditions: no extra weight, with keys, with a mobile phone, and with a bottle of water. Impact acceleration and spatio-temporal parameters were analysed through a wireless triaxial accelerometry system composed of three accelerometers: two placed in each tibia and one placed on the forehead. A higher tibia acceleration rate in the dominant leg was observed when participants ran holding both a mobile phone (*p* = 0.027; ES = 0.359) and a bottle of water (*p* = 0.027; ES = 0.359), compared to no extra weight. No changes were observed in peak acceleration, acceleration magnitude, and shock attenuation in any other conditions. Likewise, neither stride frequency nor step length was modified. Our results suggest that recreational runners should not worry about carrying objects in their hands, like a mobile phone or a bottle of water, in short races because their effect seems minimal.

## 1. Introduction

Nowadays, running is one of the most practiced physical activities globally [1]. The reasons for its popularity are not only the health benefits (physical, psychological, and social) related to the practice of this activity [2] but also the low cost and the easy use of the equipment needed [3]. This is exemplified by the high increase in recreational participants observed in different marathons [4,5]. However, injuries related to running are also common [2]. According to Francis et al. [6], the annual incidence of injuries in runners is 42.7%. Although acute injuries in runners are frequent, most running injuries are caused by overuse [7] due to the cyclical and repetitive nature of running [8]. As a result, a temporary or permanent interruption of exercise (and even inability to work) because of injuries can occur, leading in many cases to the need for medical treatment, of which direct costs may even exceed EUR 1300 [9]. For this reason, the running technique is important not only to improve running economy but also to increase movement efficacy, which is related to the probability of suffering injuries [10,11]. In this sense, it has been suggested that the swinging motion of the arms is related to stability and balance during human locomotion [12], and a unilateral arm swing restriction during running may influence the injury risk caused by defective lower extremity mechanics [13].

Some amateur runners usually run carrying various implements in their hands (like keys, a mobile phone, or a bottle of water). Professional athletes (especially in long-distance competitions) should maintain an appropriate nutritional and fluid intake to avoid performance reductions and/or medical problems [14]. Thus, they usually carry fluids and nutritional supplements in competitions and during their training sessions. In this context, several studies analysed the effects of carrying additional weight on the upper limb on kinetic and kinematic variables during running [15] and walking [16,17]. Vincent et al. [15] analysed the kinematic effects of running with different handheld water bottles carried in the right hand. They found smaller maximal angles in ankle flexion, knee extension, hip extension, and knee adduction, and larger maximal angles in ankle eversion and hip adduction on the left side than on the right side. Regarding the kinetic effects, they also found greater ground reaction forces in all the water carriage conditions compared to control conditions; the minimum hip flexion moments were consistently greater on the right side than the left side. Yang et al. [17] reported that walking with an additional unilateral arm weight increased cadence and gait speed. Fowler et al. [16] observed that walking with a bag containing a load carried over one shoulder resulted in thoracic and lumbar adjustments. Thus, it seems clear that adding weight means adding resistance to the body that may restrict movements when this weight is too heavy [17]. However, walking with a lighter additional weight, which is often asymmetric, results in an overcompensation with the whole body in healthy subjects [16,17]. Although the effects of different handloads on kinetic and kinematic variables during running have been analysed previously [15], as far as we know, there are no studies that analyse the effects on impact accelerations.

During running, the rapid deceleration of the leg and foot at ground contact results in a shock wave that is transmitted through the whole body, from the foot to the head [18], measured as impact accelerations via skin-mounted accelerometers [19]. These impacts are internally attenuated thanks to the body’s passive structures (such as bones, cartilage, and ligaments), but also by active adjustments (such as eccentric muscle actions and joint angular displacements) [20]. The impacts during running have been widely studied, withaccelerometry being one of the most used techniques to register this mechanical stress in sports and physical activities [20,21,22,23,24]. This method is based on the use of low-mass accelerometers (triaxial or uniaxial), commonly placed on the tibia and the front of the head [18]. It registers in gravities (1 g = 9.8 m/s^2^) the acceleration/deceleration of the body segments to calculate the magnitude and attenuation of impact [20,21,22,23,24].

Thus, knowing that high impact acceleration values are an important factor in runners’ overuse injuries [25,26], it is worth knowing how an added weight on a hand could affect these impacts. Close to running speeds (2.22 m/s), Encarnación-Martínez et al. [27] showed that impact accelerations during Nordic walking are higher in the tibia (12%) and head (21%) than in normal walking. In addition, mechanical models showed how adding mass to the upper body could greatly affect the second peak in the ground reaction force [28]. Thus, an added arm weight could also modify the normal running pattern and, consequently, impact accelerations could also be affected.

Therefore, this study aimed to analyse the acute effects of carrying different objects in the hand on impact accelerations and spatio-temporal parameters during running. Based on previous studies [15,28], we hypothesised that: (I) running holding both a mobile phone and a bottle of water, compared to keys and no extra weight, would increase the values of all the impact acceleration variables; (II) any handload would not modify step length and stride frequency.

## 2. Materials and Methods

### 2.1. Participants

Nineteen healthy male recreational runners (age 24.3 ± 6.8 y, height 1.75 ± 0.06 m, body mass 68.1 ± 8.8 kg, training volume of 25 ± 7.38 km/week), with experience in treadmill running, participated in this study. All participants ran a minimum of twice a week in the previous year and had no injuries in the previous six months. Participants were informed about the study characteristics, and all of them provided their written informed consent. All the experimental procedures followed the Declaration of Helsinki principles and were approved by the Ethics Committee of the university (registry number: 1252703).

### 2.2. Experimental Protocol

**Figure 1:**
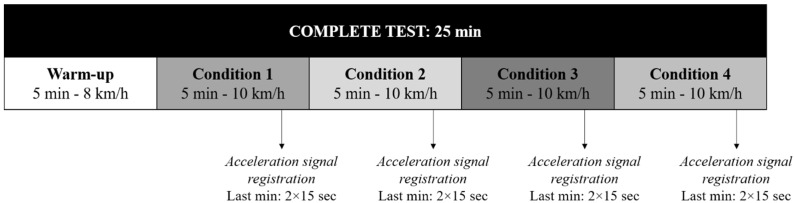
Protocol for acceleration signal registration.

In order to keep running speed constant, because it has been proved that impact accelerations can be affected by running speed modifications [18,29], the protocol was performed on a treadmill (h/p/cosmos Pulsar 3p; Traunstein, Germany). Runners were evaluated in one day. Firstly, a 5 min warm-up at 2.22 m/s (1% slope, to simulate the air resistance [30]) was performed on the treadmill. Then, 20 min of running at 2.78 m/s (1% slope) with four different conditions (5 min for each condition) was carried out (Figure 1): (A) no extra weight; (B) with keys, 0.055 kg; (C) with a mobile phone, 0.17 kg; and (D) with a bottle of water, 0.50 kg. The order of the conditions was randomly assigned and there were no breaks between them. The objects were always held in the dominant hand, and each participant decided how to hold them to feel as comfortable as possible. Additionally, rating of perceived exertion (RPE) was recorded during the last 30 s of each condition by means of a 20-point Borg scale [31].

In order to distinguish between the dominant and non-dominant leg, we used the question “If you would shoot a ball at a target, which leg would you use to shoot the ball?”, which has been shown to be a reliable assessment [32]. Additionally, to determine the dominant hand, we used the Edinburgh Handedness Inventory—Short Form [33]. All participants were both right-footed and right-handed.

Impact accelerations were registered using a wireless triaxial accelerometry system (Blautic^®^, Valencia, Spain; sampling frequency 240 Hz, range ± 16 g, mass 0.025 kg) composed of three accelerometers. Two accelerometers were placed on the distal end of each tibia [18], as it is a region with little soft tissue between the skin and the bone, with the vertical axis of the accelerometer parallel to the vertical axis of the tibia. Additionally, the third one was placed on the participant’s forehead, with the vertical axis of the accelerometer perpendicular to the ground, to measure the effectiveness of the body at attenuating the acceleration resulting from the ground contact [18]. According to the recommendations of Encarnación-Martínez et al. [21] and Lucas-Cuevas et al. [18], accelerometers were fixed to the skin with double-sided tape and neoprene tape was used to reinforce the fastening, adjusting the pressure up to the participants’ comfort limit.

The acceleration signal was registered in the last minute of each condition, during two consecutive periods of 15 s in order to reduce the error caused by the step variability [34].

### 2.3. Data Processing

A custom routine performed with Matlab R2018b (Mathworks Inc., Natick, MA, USA) was used to analyse the acceleration data. The acceleration provided by each accelerometer was corrected using a calibration file for each accelerometer and passing the acceleration on each axis through a low-pass filter (Chebyshev type II, order 8, bidirectional filter with a cut-off frequency of 50 Hz). The signal was then segmented by calculating the signal period (using the autocorrelation) and locating the points of interest (maximum, minimum, etc.), respectively, for each step [35].

Impact acceleration parameters—peak acceleration on the head and tibias (maximal acceleration value), acceleration magnitude (difference between maximal and minimal acceleration values), acceleration rate (acceleration slope, taking as extremes the moments associated with 20% and 80% of the amplitude between the minimum and the maximum) [36], and shock attenuation (reduction in peak acceleration from the tibia to the head)—as well as spatio-temporal parameters (step length and stride frequency), were analysed from the acceleration signal data of the vertical axis, detecting heel strikes in each leg.

### 2.4. Statistical Analysis

Statistical analysis was performed using SPSS 25.0 (IBM Armonk, New York, NY, USA). Descriptive statistics were described as the means ± standard deviation (SD). Normality and homoscedasticity were checked by the Shapiro–Wilk test and Levene test, respectively. As inferential analysis, impact characteristics, spatio-temporal parameters, and perceived exertion among different conditions were compared by a repeated measures ANOVA with 2 within-subjects factors (leg and handload) or a non-parametric alternative (Friedman test). As significant statistical differences in a non-parametric variable were found, the post hoc Wilcoxon test was carried out in order to explore the effects of each interaction between the different objects held in the hand. Effect size (ES) was assessed using Cohen’s *d* (≥0.2, small; ≥0.5, moderate; ≥0.8, large) [37] for parametric data and Rosenthal’s *r* (≥0.1, small; ≥0.3, moderate, ≥0.5, large) [38] for non-parametric data. Significance was defined as *p* < 0.05 and moderate to high ES (*d* ≥ 0.5; *r* ≥ 0.3).

## 3. Results

Table 1 presents descriptive data of impact acceleration and spatio-temporal parameters as well as the *p*-values of each factor (leg and handload) and their interaction from the repeated measures ANOVA model. Moreover, *p*-values from the Friedman test (for non-parametric variables) are also indicated. As is shown, higher tibia acceleration rates in the dominant leg in the conditions “with a mobile phone” (*p* = 0.027; ES = 0.359) and “with a bottle of water” (*p* = 0.027; ES = 0.359) compared with “no extra weight” were observed. No differences were found in the rest of the impact acceleration variables between the different handload conditions (*p* > 0.05; Table 1).

Regarding spatio-temporal parameters (Table 1), although significant differences were reported in step length in the leg factor (*p* = 0.008), no differences were found in the handload factor nor in the leg×handload interaction. Additionally, no differences were found in stride frequency. Finally, regarding perceived exertion, no significant differences were found among any conditions (*p* = 0.972): no weight, 11.26 ± 2.40; keys, 11.30 ± 2.15; mobile phone, 11.30 ± 2.66; bottle of water, 11.41 ± 2.71.

## 4. Discussion

Running is one of the most common forms of exercise, especially as a recreational activity [39]. Among amateur runners, it is very common to practice this activity carrying different objects in the hand, like a mobile phone or a bottle of water. This study aimed to analyse the effects of carrying different objects in the hand on impact accelerations and spatio-temporal parameters during running. The main result was that tibia acceleration rate increased (with a moderate effect size, *r* ≥ 0.3) in the dominant leg when participants held a mobile phone or a bottle of water compared to running with no extra weight. Meanwhile, no differences were found in the rest of the impact acceleration variables, nor in spatio-temporal parameters among the different handload conditions.

Prolonged exposure to high acceleration rates and magnitudes during long distance running has been associated with an increased injury rate because the musculoskeletal system is less effective in attenuating these impacts at the end of a race due to fatigue [25,40]. For this reason, impact acceleration analysis has gained attention for the assessment of equipment [21,41], training [11], or running technique [42]. Moreover, Pérez-Soriano et al. [11] explained that not only a high impact acceleration peak, but also high levels of acceleration rate, could increase the probability of injuries during running. This is because an impact that is transmitted more quickly may be more difficult to attenuate than one of the same magnitude that is transmitted more slowly [11,43]. In this sense, Milner et al. [25] found a strong correlation between loading rates and tibial shock, so that runners with a history of stress fractures showed a higher acceleration rate than uninjured athletes. In our study, as mentioned above, acceleration rate of the dominant tibia increased when participants ran holding a mobile phone and a bottle of water, but not when holding keys. However, no changes were reported in peak acceleration, acceleration magnitude, and shock attenuation in any condition. Therefore, the hypothesis that running holding both a mobile phone and a bottle of water, compared to keys and no extra weight, would increase the values of all the impact acceleration variables was rejected.

Liew et al. [44] showed that running with a backpack load of 20% of the body weight changes ankle, knee, and hip angles, which would support the idea that runners adjust their lower-extremity technique to cope with the added weight [15]. Consequently, impact acceleration levels would also be modified, because changes in running technique modify impact acceleration [11]. However, changes found by Liew et al. [44] were produced when running at 4 and 5 m/s, but not when participants ran at 3 m/s, which is a speed close to our study speed. Furthermore, it must also be noted that 20% of the body weight is heavier than the weight used in our study. According to Yang et al. [17], adding extra weights leads to additional resistance on the body and could restrict movements when the added weights are too heavy. Nevertheless, when these extra weights are lighter, healthy subjects are able to overcome them. Thus, it seems that both the running speed and additional weight used in our experimental protocol were not high enough to find changes in these variables. On the other hand, previous studies have compared the dominant and non-dominant leg, finding differences in other biomechanical variables, like range of motion in the knee and hip joints, peak ground reaction force, and loading rate [45], or lower leg angle, rearfoot angle, and velocity of the heel at touchdown [46]. Similarly, our results also showed differences between the dominant and non-dominant leg, with a higher acceleration rate in the dominant leg (with a moderate effect size) when participants ran holding both a mobile phone (+16.05%) and a bottle of water (+13.40%) compared to running without weight. However, no differences were found in the keys condition, perhaps because the keys’ weight was not high enough to provoke alterations in impact accelerations. Consequently, the changes found in our study in the acceleration rate of the dominant tibia should be interpreted cautiously, and future studies with higher running speed and handload weight will be necessary to verify this increase.

Regarding spatio-temporal variables, none of them was modified in any condition. Therefore, the hypothesis that step length and stride frequency would not be modified by any handload was accepted. Vincent et al. [15], with a similar running protocol (20 min in total at 4 m/s), also found no differences in these two variables. This could be because runners make slight adjustments in their lower-limb technique in order to overcome small loads (full water bottle, 0.454 kg; half-full water bottle, 0.227 kg) [15]. In our study, running intensity and duration were not high in order to avoid fatigue in the runners (the perceived exertion of our sample was “fairly light” [31]), which has been shown to cause changes both in stride frequency and step length [47]. Additionally, amateur runners show greater kinematic changes during a fatigued run, and the injury probability could also be higher [48]. Therefore, future investigations should analyse the effects of asymmetric handloads in fatigued running (or at least more intense running).

As a practical application of this study, as the only differences were found in tibial acceleration rate, and the effect size was not large, we believe that we can suggest that recreational runners should not worry about carrying objects like a mobile phone or a bottle of water on their hand in short races, because their effect seems to be minimal. However, our study has not evaluated the effect in a long-distance race, so in these cases it would be preferable to be cautious and to avoid handload (if hydration is guaranteed during the race), while future studies should evaluate if the impact acceleration of the tibia increases as the duration increases. We are, of course, aware that avoiding additional weight is not always possible (e.g., if runners must manage hydration themselves), so future studies should evaluate the effects of different amounts of added weight on impact acceleration during running using different supports, like a backpack, a vest pack, or a waist belt, instead of using the hands.

Finally, this study had some limitations. Firstly, although it was not the aim of our study, running time and velocity were not enough to generate fatigue, which produces kinematic changes [47,48]. Therefore, a longer running time would have been necessary to check if fatigue produces different modifications in impact acceleration variables and spatio-temporal parameters during running with a handload. Secondly, while our study was focused on the acceleration impact analysis in the lower limbs, a kinematic analysis of arm swinging could provide more information to identify other causes of the changes found. Thirdly, this study was carried out on a treadmill, so a more ecological approach to running in a real context would also be interesting.

## 5. Conclusions

In conclusion, running holding both a mobile phone and a bottle of water in the hand produces a moderate increase in the acceleration rate in the dominant leg, with no effects on other impact acceleration variables. No changes in stride frequency or step length were found in any condition, nor in the rating of perceived exertion. Thus, based on our results, recreational runners should not be worried about carrying objects in their hands, like the ones used in our study, in short races because their effect seems to be minimal.

## Figures and Tables

**Table 1:** Analysis of impact acceleration and spatio-temporal parameters.

	No Weight	Keys	Mobile Phone	Bottle of Water	*p*-Value (Parametric)			*p*-Value (Non-Parametric)
					Leg	Handload	Leg × Handload	
Head peak acceleration, D (g)	2.05 ± 0.46	2.05 ± 0.43	2.06 ± 0.40	2.03 ± 0.42	0.183	0.585	0.432	-
Head peak acceleration, ND (g)	2.02 ± 0.48	1.98 ± 0.43	2.02 ± 0.43	1.98 ± 0.42				
Tibia peak acceleration, D (g)	6.13 ± 1.39	6.14 ± 1.35	6.22 ± 1.36	6.32 ± 1.42	-	-	-	0.776
Tibia peak acceleration, ND (g)	6.13 ± 1.51	6.20 ± 1.50	6.27 ± 1.42	6.23 ± 1.47				0.500
Head acceleration magnitude, D (g)	2.18 ± 0.49	2.17 ± 0.45	2.19 ± 0.44	2.16 ± 0.45	0.133	0.470	0.723	-
Head acceleration magnitude, ND (g)	2.14 ± 0.48	2.10 ± 0.45	2.14 ± 0.47	2.10 ± 0.44				
Tibia acceleration magnitude, D (g)	6.12 ± 1.36	6.16 ± 1.33	6.26 ± 1.31	6.35 ± 1.35	-	-	-	0.822
Tibia acceleration magnitude, ND (g)	6.07 ± 1.51	6.16 ± 1.49	6.22 ± 1.40	6.22 ± 1.49				0.730
Head acceleration rate, D (g/s)	68.39 ± 19.80	69.89 ± 21.55	69.72 ± 18.44	70.19 ± 20.52	0.428	0.964	0.327	-
Head acceleration rate, ND (g/s)	68.29 ± 17.98	67.01 ± 16.25	67.95 ± 15.77	66.39 ± 15.83				
Tibia acceleration rate, D (g/s)	247.39 ± 104.31	262.27 ± 117.22	287.09 ± 126.15 *	280.55 ± 118.41 *	-	-	-	**0.006**
Tibia acceleration rate, ND (g/s)	296.28 ± 147.65	301.71 ± 145.68	298.22 ± 144.43	290.12 ± 131.37				0.938
Shock attenuation, D (%)	65.88 ± 7.57	65.86 ± 7.93	66.15 ± 7.58	66.98 ± 7.81	0.984	0.495	0.622	-
Shock attenuation, ND (%)	65.74 ± 9.86	66.35 ± 9.57	66.12 ± 9.94	66.54 ± 9.44				
D step length (m)	0.93 ± 0.09	0.94 ± 0.09	0.94 ± 0.08	0.93 ± 0.07	**0.008**	0.960	0.986	-
ND step length (m)	0.86 ± 0.10	0.86 ± 0.10	0.85 ± 0.11	0.86 ± 0.11				
Stride frequency (Hz)	1.56 ± 0.09	1.56 ± 0.10	1.56 ± 0.09	1.56 ± 0.10	-	0.958	-	-

Mean ± SD; bold means *p* < 0.05; * difference from no weight (*p* < 0.05 and *r* ≥ 0.30), from post hoc Wilcoxon test; D = dominant leg; ND = non-dominant leg.

## Data Availability

The dataset generated and analysed during the current study are available from the corresponding author on reasonable request.
